# Research Progress on the Application of Nanopore Long-Read Sequencing Combined with DNA Methylation Marking in Complex Forensic Evidence

**DOI:** 10.3390/genes17101235

**Published:** 2026-10-07

**Authors:** Qiannan Xu, Xuanman Cui, Shuyan Mei, Haojie Qin, Zhe Zheng, Xiandun Zhai

**Affiliations:** 1 School of Basic Medicine and Forensic Medicine, Henan University of Science and Technology, Luoyang 471023, China; xqn2550038357@163.com (Q.X.);; 2 Institute of Medical Aspects of Specific Environments, Henan University of Science and Technology, Luoyang 471023, China; 3 Forensic Medicine Identification Center, Henan University of Science and Technology, Luoyang 471023, China

**Keywords:** nanopore long-read sequencing, DNA methylation, complex forensic evidence, personal identification, epigenetic markers

## Abstract

Background In the analysis of complex forensic material evidence, conventional detection methods for highly degraded, trace, or mixed samples present several limitations, such as low typing success rates and limited information dimensions. Methods This study systematically examines the technical principles and compatibility of nanopore long-read sequencing combined with DNA methylation profiling, identifies optimization strategies to enhance sensitivity for trace materials, improves accuracy in methylation site interpretation, for instance, NanoFreeLunch, without raw signals and using base quality/error patterns, reaching 0.97–0.99 correlation with benchmark regional methylation levels, and streamlines library preparation workflows while evaluating its practical efficacy through domestic and international case studies. Results The research demonstrates that this technology enables simultaneous acquisition of genomic sequences and epigenetic information, facilitating multidimensional identification—including personal identification, age estimation, and body fluid identification—even in highly degraded samples, with significantly higher site detection rates compared to traditional methods. Conclusions Future efforts should focus on standardizing the technology and developing specialized databases to further refine its application frameworks for forensic investigations, thereby providing robust technical support for complex forensic evidence analysis.

## 1. Introduction

Forensic identification plays a pivotal role in case resolution and judicial determination. However, highly degraded, trace, and mixed samples remain fundamental challenges that limit the efficacy of traditional techniques during practical investigations. Conventional methods such as short tandem repeat (STR) typing, real-time polymerase chain reaction (qPCR) quality assessment, mitochondrial DNA (mtDNA) sequencing, and conventional PCR amplification, while well-established for routine samples, exhibit significant limitations—including excessive reliance on long fragments, instability with low template quantities, limited information dimensions, and difficulty integrating sequence and epigenetic data—resulting in insufficient personal identification capacity and extended inference capabilities for complex specimens. The advancement of nanopore long-read sequencing technology, which enables simultaneous reading of sequences and DNA methylation information without PCR amplification, offers a novel technical approach for analyzing challenging forensic samples. Particularly in age estimation, body fluid identification, and personal identification, methylation markers demonstrate substantial potential for forensic applications. Therefore, a systematic analysis of the integration between nanopore long-read sequencing and forensic-specific methylation markers—covering their technical principles, compatibility, optimization strategies, and standardization prospects—not only addresses the bottlenecks in identifying highly degraded samples but also provides theoretical foundations and practical guidance for transitioning forensic evidence analysis from single-dimensional typing to multidimensional comprehensive interpretation.

Literatures were identified via PubMed, Web of Science, Scopus, China National Knowledge Infrastructure (CNKI), Wanfang data, and Google Scholar up to July 2026, using keywords including nanopore/long-read sequencing, DNA methylation, forensic genetics, complex/degraded/trace evidence, body fluid identification, age estimation, and STR typing. We included original studies, reviews, and dissertations directly relevant to nanopore sequencing and/or DNA methylation markers in forensic complex evidence; clinical or methodological papers were included only when transferable. Duplicates, non-forensic papers, and reports lacking methodological detail were excluded. Reference lists of key reviews were screened. This was a narrative, not systematic search, so completeness may be limited.

## 2. Current Technical Bottlenecks in the Identification of Highly Degraded Forensic Samples Using Challenging Methods

In contemporary forensic evidence identification practice, challenging samples such as those with high degradation, trace amounts, or mixed compositions have become significant application barriers that conventional techniques struggle to overcome. Traditional STR typing techniques based on capillary electrophoresis rely on the amplification of specific length fragments; however, when samples undergo severe degradation due to environmental exposure, the amplification success rate of long fragment alleles significantly declines. Even when miniSTR designs are employed to shorten the amplification fragments, limitations in the number of fluorescent channels prevent the inclusion of sufficient effective loci, rendering the typing results inadequate for personal identification [1,2]. Traditional capillary electrophoresis (CE)-based STR profiling remains the gold-standard forensic identification method, yet it suffers from poor performance for highly degraded DNA and limited capacity for parallel detection of single-nucleotide polymorphisms (SNPs), indels and epigenetic markers. Short-read massively parallel sequencing (MPS) partially mitigates these drawbacks: it enables high-throughput multiplex detection of different markers from fragmented forensic templates, improving mixture deconvolution and degraded-sample genotyping [3,4]. Nevertheless, short-read MPS cannot directly capture native DNA methylation and struggles with highly repetitive genomic regions. By contrast, Oxford Nanopore Technology (ONT) long-read sequencing preserves full-length DNA fragments, permits direct epigenetic modification reading, and provides extra analytical value for challenging forensic specimens.

Specialized detection technologies such as sex determination using pyrosequencing can detect short fragments as small as 45 bp but only provide single-dimensional identity information, failing to achieve comprehensive individual tracing and often encountering issues of “invalid amplification” caused by Amelogenin (AMEL) sequence mutations or sex chromosome variations [5]. Conventional next-generation short-read sequencing technologies also depend on PCR amplification, which completely loses epigenetic information such as DNA methylation, thereby failing to meet the demands of advanced identification tasks like age estimation [6].

The pre-processing of complex forensic samples faces significant challenges (Table 1). Conventional DNA extraction methods such as the Chelex-100 method and magnetic bead technique often fail to completely remove inhibitors or minimize DNA degradation when handling hard tissues or decomposed specimens, thereby reducing the success rate of subsequent analyses [7,8,9]. Existing DNA quality assessment tools like qPCR exhibit poor stability and limited applicability to complex samples, failing to provide precise quantitative data for optimizing detection protocols. Although mitochondrial DNA sequencing can be applied to severely degraded samples, it only enables maternal lineage tracing with insufficient personal identification resolution and cannot serve as sole conclusive evidence [10]. Emerging technologies like microbiome profiling still face challenges including dynamic variability and inadequate data standardization, falling short of forensic application standards [11]. Conventional PCR techniques demonstrate strong bias toward amplifying low template quantities and are susceptible to contamination, further limiting their efficacy in microsample analysis [12,13]. These technical limitations collectively increase failure rates in complex sample identification, rendering numerous critical forensic evidence items ineffective during criminal investigations [14].

Forensic genetics currently relies on CE-based STR analysis, increasingly complemented by MPS, mainly Illumina short-read platforms. MPS overcomes CE limitations by enabling high-throughput multiplexing, sequence-based allele calling, and expanded marker panels. Nevertheless, short reads poorly resolve long repeats, allele phasing, structural variants, and endogenous DNA methylation. ONT long-read sequencing offers kilobase-length reads, real-time analysis, portability, and direct methylation detection, benefiting complex, degraded, mixed, and trace forensic evidence, full mitochondrial genomes, and species identification, although accuracy, throughput, and bioinformatics still limit routine implementation.

## 3. Technical Principles and Compatibility of Nanopore Long-Read Sequencing Combined with DNA Methylation

### 3.1. Technical Mechanism of Nanopore Long-Read Sequencing for Simultaneous Acquisition of Sequences and Methylation Information

The fundamental operating principle of nanopore long-read sequencing involves utilizing single-molecule nanopore sensing technology to detect changes in current intensity, frequency, and periodicity during nucleic acid molecules passing through the nanopore, thereby enabling sequence determination. Notably, this method can simultaneously obtain information on base modifications such as DNA methylation without requiring PCR amplification or bisulfite conversion [15]. Methylation modifications alter the electrical charge properties of DNA bases, resulting in distinct electrical signal characteristics when passing through nanopores compared to unmodified bases; establishing decoding models based on these signals allows for precise identification of methylation sites. Studies have demonstrated that the use of R10.4 flowcell reagents [16] and optimized base recognition algorithms further enhances methylation detection accuracy. The development of novel approaches—including the PoreFormer model employing hierarchical clustering and attention mechanisms for current signals, and the NanoFreeLunch method that operates independently of raw electrical signals—has provided robust technical support for accurate interpretation of diverse methylation patterns. Specifically, the NanoFreeLunch method achieves correlation coefficients between its genomic average methylation level predictions and reference methods ranging from 0.97 to 0.99 [17,18]. Traditional bisulfite sequencing has limitations, such as susceptibility to DNA degradation and generation of short-read fragments, whereas nanopore sequencing enables direct long-read sequencing of native DNA molecules. Studies have successfully achieved sequencing of natural DNA reads extending up to thousands to tens of thousands of base pairs, or even up to megabase (Mb) scale [19,20,21]. Additionally, nanopore sequencing is well-suited for low starting sample quantities and highly degraded samples. The RCnano method, based on the adapter extension strategy, enables approximately sixfold higher sequencing output compared to standard library preparation workflows and can detect methylation sites at abundance levels as low as 0.1% [20]. Its requirement for no complex sample pretreatment also facilitates simplified library preparation procedures for forensic specimens [21,22]. Furthermore, its single-molecule detection capability lays the foundation for simultaneous analysis of methylation and sequence information at the single-cell level [23].

### 3.2. Screening and Application Value of Forensic-Specific Methylation Markers

The screening of forensic-specific methylation markers must meet several critical criteria: the sites must remain stable in degraded samples, exhibit specificity across individuals, demonstrate strong relevance to forensic identification targets, and be compatible with the long-read sequencing capabilities of nanopore technology to prevent site loss due to fragment fragmentation. Currently, well-established methodologies exist for genome-wide identification of methylation markers, such as the differential methylation library construction approach combining methylation-sensitive restriction enzyme digestion with inhibition-based differential hybridization, which efficiently identifies specific methylation patterns across diverse scenarios and provides methodological guidance for forensic marker discovery [24]. For age estimation applications, existing studies have identified multiple CpG sites strongly associated with age; a domestically developed digital droplet PCR (dd PCR)-based model based on seven core CpG sites achieved an average absolute deviation of 3.45 years in ten-fold cross-validation without significant gender-related differences, providing a reliable marker foundation for forensic age determination [25]. For body fluid identification, loci such as *DACT1* and *USP49* are suitable for semen analysis, while *cg26107890* and *cg20691722* enable differentiation between saliva and vaginal secretions, requiring only minimal DNA amounts without excessive sample consumption [26].

Beyond the conventional forensic identification scenarios mentioned above, methylation markers also hold significant potential applications in inferring individual health characteristics relevant to criminal cases. Research findings on disease-specific methylation markers can provide valuable insights for expanding forensic identification approaches. For instance, genome-wide methylation screening strategies in colorectal cancer blood testing have successfully identified multiple highly specific disease-associated biomarkers [27]. Joint detection of *SDC2* and *TFPI2* methylation enhances colorectal cancer sensitivity while maintaining specificity exceeding 90% [28]. Methylation status of *NEUROG1* in fecal samples serves as an effective adjunct indicator for detecting colorectal cancer and advanced adenomas [29]. Systematic reviews on oral cancer further demonstrate that non-invasive or minimally invasive methylation assays offer viable methods for early disease detection [30]. These research methodologies and validation frameworks for disease-specific methylation markers can serve as references for identifying biomarkers for individual health characterization in forensic contexts.

Nanopore adaptive sampling technology enables simultaneous detection of age-related and body fluid-specific methylation markers within a single sample without requiring specialized sample pretreatment. Its results demonstrate high consistency with whole-genome bisulfite sequencing and can identify novel age-related methylation sites [31]. While traditional pyrosequencing allows for quantitative analysis of methylation, it necessitates customized detection protocols for specific sites and requires large sample volumes, making it inadequate $R^{2}=0.91$ for multi-marker parallel analysis of complex specimens [32]. Studies on *MGMT* promoter region methylation have shown that nanopore sequencing and pyrosequencing yield comparable results, covering all 98 CpG sites in the region, thereby providing technical evidence for expanding the application of methylation markers in forensic contexts [33]. Nanopore sequencing workflow for forensic methylation marker analysis was showed in Table 2 and Table 3.

## 4. Optimization Pathways for Technical Challenges in Handling Highly Degraded Specimens

### 4.1. Improvement Plan for Enhanced Sensitivity in Microsample Detection

**Figure 1:**
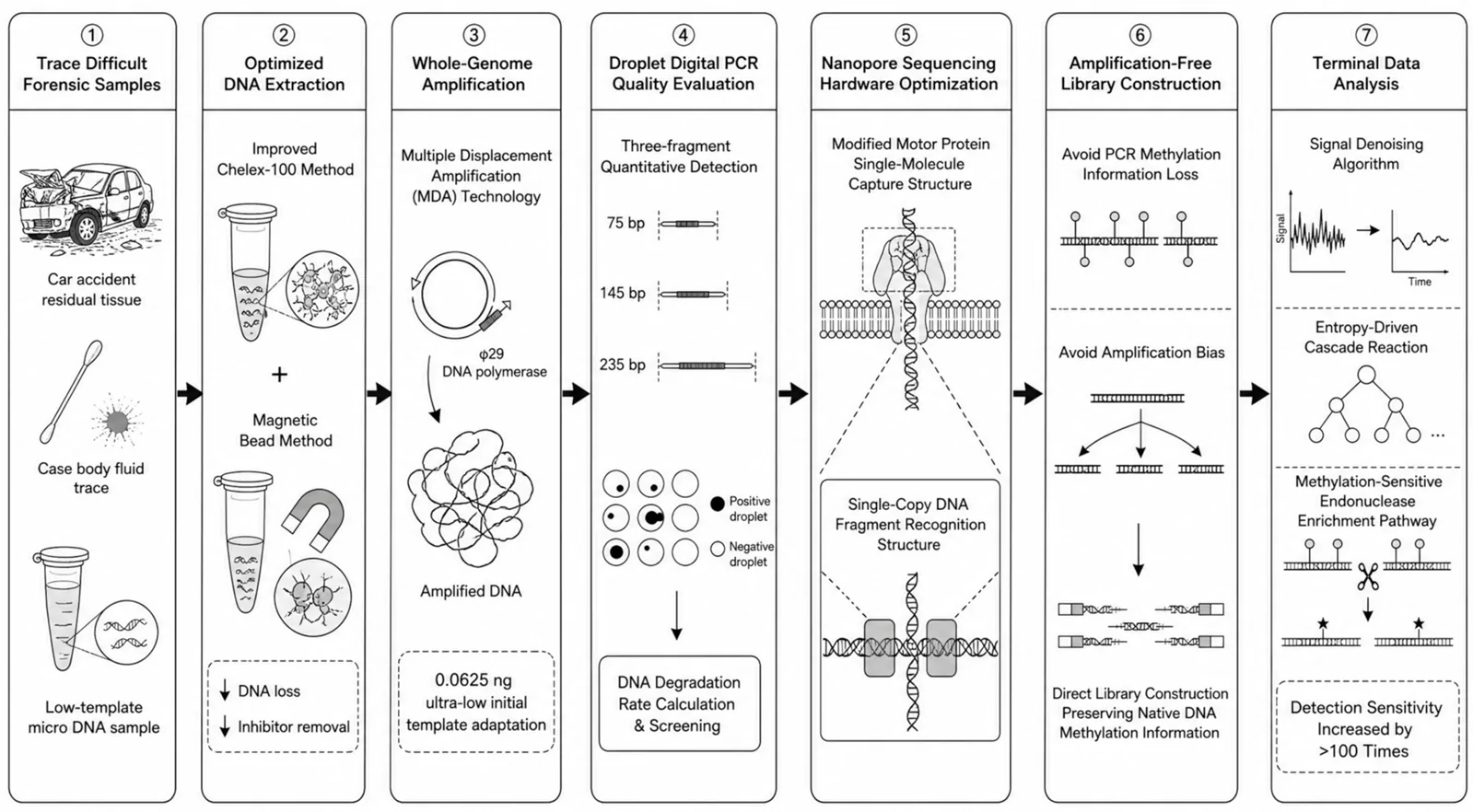
Improvement Scheme for Detection Sensitivity of Trace Materials.

To meet the detection requirements for trace-level challenging samples at the picogram (pg) level, such specimens represent common subjects of complex analysis in forensic evidence identification practice and possess significant diagnostic value in practical cases such as vehicle identification in traffic accidents and traceability of body fluids in sexual crime investigations [34]. Current technical approaches enhance sensitivity through multi-step optimization: the library preparation stage can incorporate whole-genome amplification techniques such as multiplex replacement amplification, which have been proven effective in reducing amplification bias in low-template samples and meeting detection demands starting from 0.0625 ng of template [35]. Concurrently, targeted improvements to extraction methods like Chelex-100 and magnetic bead techniques minimize DNA loss and inhibit inhibitor residues [36]. Combined with a quality assessment protocol based on microdroplet-based digital PCR, precise screening of trace samples suitable for sequencing is achieved through quantitative analysis of three fragments (75 bp, 145 bp, and 235 bp) and calculation of degradation rate parameters. In terms of sequencing hardware, the DNA-binding activity of nanopore motor proteins can be modified to enhance single-molecule capture efficiency, meeting the requirements for identifying single-copy-level DNA fragments. Concurrently, amplification-free library preparation strategies tailored for microsample analysis have been developed to prevent loss of methylation information and amplification bias during PCR [37], though this is not specifically for nanopore sequencing. For data analysis, signal-to-noise ratio reduction algorithms can be optimized to filter out low-quality sequencing signals, improving the accuracy of sequence and methylation site detection even with low coverage data. By combining molecular detection strategies such as entropy-driven cascade reactions and methylation-sensitive endonuclease enrichment, the detection sensitivity of target methylation regions can be increased by more than 100-fold [38] (Figure 1).

Whole-genome amplification and native methylation analysis are indeed not readily compatible. We recommend whole-genome amplification solely for low-template samples when the aim is sequence recovery, such as STR typing or species identification; it should not be used when native methylation information is required. Conversely, amplification-free nanopore preparation is essential for direct methylation calling. These two objectives therefore require separate workflows and cannot be achieved simultaneously from the same amplified template.

### 4.2. Optimization Strategies for Methylation Site Interpretation Accuracy

Highly degraded samples have issues with methylation signal deviations, and mixed samples have problems with typing interference. These can be improved by optimizing multi-dimensional algorithms and using database support to enhance the accuracy of interpretation. Firstly, the PoreFormer model based on nanopore current signal stratification clustering and attention mechanism can be used to adapt to multiple types of methylation modifications and sequence diversity scenarios, thereby reducing the noise interference caused by degradation of the signals. At the same time, the NanoFreeLunch method that does not require the original signal can be introduced, relying on base quality values and sequencing error patterns for modeling, to achieve quantitative methylation detection of public sequencing data. The correlation between this method and the benchmark method’s regional average methylation level can reach 0.97 to 0.99. Considering the typing requirements of mixed samples, a mixed unsupervised clustering method that combines base information and original current signals can be used to improve the splitting accuracy of short-read fragments and barcode samples, and reduce signal cross-interference from different sources of samples [39]. Based on this, a forensic-specific methylation reference database is established, incorporating methylation profiles from different degradation levels and different tissue sources as correction benchmarks. Combined with the R10.4 flow cell chemical mode and the optimized base recognition algorithm, the consistency and accuracy of site interpretation are further improved. For highly degraded special samples like formalin fixed paraffin-embedded (FFPE) tissues, the existing methylation detection quality control logic based on a high-resolution melting (HRM) curve can be referenced, and the DNA quality of the samples can be pre-assessed and graded [40], matching the corresponding weight signal correction parameters. For trace cell-free DNA (cfDNA) samples, the single-strand library method is preferred to improve the capture efficiency of methylation molecules, providing a high-quality sequencing data foundation for subsequent interpretation [41]. And based on the verified STR typing algorithm’s error correction logic, a systematic deviation correction model for methylation sites is established to reduce the impact of sequencing inherent errors on the results [42].

Although nanopore long-read sequencing offers real-time, amplification-free detection and preserves native methylation, severe degradation inherently limits intact long molecules. Short fragments lower pore utilization and data quality, making results strongly fragment-length-dependent and requiring higher minimum input. Coverage then becomes uneven, and achieving sufficient depth may demand more DNA, directly trading off against retention of native methylation. For highly fragmented forensic samples, researchers should report input thresholds and fragment-size distributions, acknowledge coverage loss, and consider complementary short-read or overlap-extension PCR approaches. Conclusions should therefore remain cautious when DNA is trace, mixed, or severely degraded.

### 4.3. Simplification Plan for Sequencing Library Construction Process

To address the need for rapid on-site identification of complex biological samples, existing simplified library preparation strategies have achieved multidimensional technological breakthroughs. The transposase-based rapid library preparation method can replace traditional ligase-based approaches, significantly reducing operational steps and labor costs while meeting the full-process requirements of portable detection systems [43]. For methylation analysis of minimally degraded samples, single-stranded cfDNA library preparation demonstrates higher molecular utilization efficiency compared to conventional double-stranded methods, making it more suitable for methylation studies. For challenging samples requiring simultaneous multi-omics analyses, the digital microfluidic-based DIRECT technology enables shared library preparation without separation of single-cell DNA methylation profiles and transcriptomes, reducing sample loss while simultaneously elucidating cellular gene regulatory networks—a viable approach for multidimensional analysis of precious micro samples [44]. The MspI enzyme digestion-preprocessed whole-genome methylation high-throughput sequencing library construction method enhances targeted capture efficiency of methylation sites while further lowering library preparation barriers for minimally degraded samples [45]. Compared to standard nanopore-based libraries, the RC nano library preparation workflow using RC adapters achieves approximately sixfold higher sequencing yield and additionally recovers 42% of unclassified reads through secondary sample splitting, significantly reducing initial template requirements [46]. For on-site tracing of unknown pathogens during public health emergencies, the integration of nanopore adaptive sampling technology enables targeted enrichment of target pathogen sequences during sequencing, significantly enhancing the efficiency of field detection for emerging infectious diseases such as monkeypox [47]. The accompanying mobile analysis tool Genopo facilitates migration of nanopore sequencing data analysis workflows to Android devices, allowing for on-site interpretation of information such as methylation profiles without relying on laboratory computing resources [48].

## 5. Technical Application Prospects and Standardization Directions

Currently, this technology still faces many obstacles during its popularization process. In the field of forensic medicine, insufficient computing power support, poor data sharing, low algorithm transparency, and limitations on infrastructure construction of institutions are some common problems [49]. Moreover, nanopore sequencing itself has defects. Its data analysis process is relatively complex, and the original sequencing error rate is relatively high. In the application of RNA modification detection, microbial methylation recognition, etc., there are also problems such as imperfect algorithm models, massive size of raw pod5 files and inconsistent detection processes that need to be solved. In the digital forensics field, there are challenges such as rapid technological iteration and imperfect evidence validity determination rules [50]. Apart from the methylation detection technology related to nanopore sequencing, numerous emerging technologies such as microhaplotype, lateral flow immunoassay DNA recovery, forensic sporeology, Raman spectroscopy, DNA barcode, soil trace evidence analysis, forensic imaging, and methylated derivative detection of butyl tin compounds in sediment also show broad application potential in forensic evidence identification, providing more technical approaches for the multi-dimensional examination of difficult evidence. To clarify the maturity of each technology in forensic medicine, Table 4 summarizes the different application types and verification stages based on nanopore sequencing and methylation markers, and marks the key considerations such as error rate, repeatability, contamination risk, mixed-sample deconvolution, inter-laboratory validation, population effect, and evidence threshold [50].

The implementation of related technologies also faces common problems such as the lack of detection standards and the lag in database construction [51]. The exploration of related standardization can provide references for the improvement in the model of methylation detection technology. To improve the standardization of the technical model, based on the requirements of forensic identification scenarios, the threshold for interpreting methylation sites should be unified, and the process of sequencing and library construction should be simplified [52]. A cross-institutional shared database of forensic-specific methylation markers should be established, and quality control norms for the entire detection process should be established as a supporting measure. The gradual inclusion of technical inspection results in the judicial evidence validity determination model should be promoted, thereby providing stable and reliable technical support for the identification of difficult forensic evidence.

Nanopore long-read sequencing is rapidly emerging as an integrated platform for forensic age estimation, body fluid identification, personal identification, and analysis of degraded or mixed traces. Beentjes et al. [53] evaluated PromethION 2 adaptive sampling for simultaneous epigenetic age prediction and body fluid identification from <100 ng DNA; although low coverage distorted methylation β-values and biased age estimates, a proof-of-concept linear correction improved accuracy, and blood and saliva were correctly identified in 4/4 samples. de Bruin et al. [54] demonstrated a single ONT adaptive direct-sequencing assay covering STRs, SNPs, InDels, mtDNA, and methylation markers, achieving 90–92% correct STR identification, 98% SNP identification, and complete mtDNA coverage, while low-quality samples remained challenging. For degraded traces, Nakanishi et al. [55] used nanopore sequencing to quantify DNA fragments > 10,000 bp in bloodstains and showed that the proportion of long fragments, especially >50,000 bp, decreased with storage time and after a single water exposure, linking fragment-length profiles to stain age and environmental history. Li et al. [56] developed a targeted mRNA nanopore panel containing 12 body fluid-specific mRNAs and 2 reference genes with 41 coding region SNPs (cSNPs), enabling body fluid and donor inference; specific transcripts showed >95% read proportions in peripheral blood, semen, and menstrual blood, with accurate clustering despite cross-reactivity, and discrimination power of 0.5645–0.9017. Together, these studies support nanopore sequencing as a promising tool for complex forensic evidence, but also highlight the need for validated thresholds, improved low-input and mixed-sample performance, and larger population studies.

Additionally, PacBio HiFi sequencing can produce comparable CpG methylation data without bisulfite treatment. It provides high-fidelity consensus sequences and accurate methylation detection. Still, forensic degraded samples pose practical challenges for PacBio workflows: it demands higher input and high-molecular-weight DNA, and relies on large laboratory-bound instruments. Nanopore sequencing tolerates lower-quality trace DNA and supports portable, real-time sequencing, making it more suitable for forensic trace-sample scenarios, though it requires careful calibration for methylation signal interpretation. These features should be weighed when selecting long-read epigenetic methods for forensic specimens [57].

## 6. Conclusions

Overall, nanopore long-read sequencing combined with DNA methylation profiling enables simultaneous acquisition of sequence and epigenetic information in highly degraded, trace, and complex mixed samples, effectively addressing the limitations of traditional STR, qPCR, and conventional short-read sequencing, such as restricted fragment length, insufficient information dimensions, and significant amplification bias. By optimizing sample preparation and library construction workflows, enhancing capture efficiency for low-template samples, implementing high-precision base identification and methylation interpretation algorithms, and integrating forensic-specific methylation databases with a tiered quality control system, this approach holds promise for improving detection rates, interpretation accuracy, and result stability in challenging specimens. This technology not only facilitates expanded applications such as age estimation, body fluid identification, and assessment of individual health characteristics but also provides novel insights for rapid traceability and evidence integration in specialized cases. However, nanopore sequencing still faces practical challenges including high inherent error rates, inadequate algorithmic transparency, lack of standardized protocols, and delayed development of cross-institutional databases. Future advancements must focus on standardizing detection procedures, unifying thresholds, constructing reference databases, establishing quality control standards, and aligning with forensic evidence regulations to ensure sustainable implementation and widespread adoption of this technology in forensic science.

## Figures and Tables

**Table 1:** Limitations of Current Techniques for Identifying Complex Forensic Evidence Samples.

Technology Category	Specific Limitations
Capillary electrophoresis STR typing technique	Dependent on the amplification of specific length fragments, the amplification success rate of long fragment alleles is relatively low for degraded samples. Due to limitations in the number of fluorescent channels, miniSTR lacks sufficient effective sites, resulting in typing results that fail to meet personal identification criteria.
Specialized detection technology for pyrophosphate sequencing	Only single-dimensional identity information can be obtained; under such circumstances, it is impossible to conduct comprehensive traceability of individuals, and it is also challenging to mitigate the issue of “invalid amplification” caused by AMEL sequence mutations or sex chromosome variations.
Conventional second-generation short-read sequencing technology	The reliance on PCR amplification leads to the complete loss of epigenetic information such as DNA methylation, making it inadequate for applications requiring extended identification, including age estimation.
Conventional DNA extraction methods (Chelex-100 method, magnetic bead method, etc.)	During the processing of samples such as hard tissues and necrotic tissues, incomplete removal of inhibitors occurs, coupled with a high rate of DNA degradation, thereby reducing the success rate of subsequent analyses.
DNA Quality Assessment Tool (qPCR)	It exhibits insufficient stability and poor applicability to complex samples, thus failing to provide a precise quantitative basis for selecting subsequent detection methods.
Mitochondrial DNA sequencing technology	It can only establish maternal lineage tracing, but its personal identification resolution is insufficient to serve as the sole conclusive evidence.
Emerging technologies for microbial community identification	There are issues such as dynamic changes and insufficient data standardization, and current practices have not yet met the standard requirements specified for forensic science applications.
Conventional PCR Technology	Low-temperature template samples exhibit strong bias during amplification and are highly susceptible to contamination interference, which significantly limits the detection efficiency of trace specimens.

**Table 2:** Nanopore Sequencing Workflow for Forensic Methylation Marker Analysis.

Workflow Step	Core Content/Key Points	Marker/Technical Basis	Reference
① Biological sample collection	Body fluids, degraded/trace forensic materials; no large sample amount required; trace amounts are sufficient	—	—
② Nanopore sequencing (adaptive sampling)	Long reads, compatible with degraded samples; no complex preprocessing required; enables parallel capture of multiple methylation markers from the same sample	Highly consistent with whole-genome bisulfite sequencing (WGBS) results; can discover novel age-related loci	[31]
③ Methylation detection	Reads the methylation status of CpG sites	Comparable to pyrosequencing results (full coverage of 98 CpG sites in the MGMT promoter region)	[33]
④ Marker screening and identification	Screening criteria: stability in degraded samples, individual specificity, and relevance to forensic objectives	Differential methylation library construction method (methylation-sensitive restriction enzymes + suppression differential hybridization)	[24]
⑤ Forensic phenotype inference and application	Multi-branch applications (see Table 3)	—	—

Note: WGBS is the abbreviation for whole-genome bisulfite sequencing.

**Table 3:** Forensic Application Dimensions and Representative Markers.

Application Scenario	Representative Marker/Locus	Description	Reference
Age inference	7 core CpG sites (ddPCR model)	Ten-fold cross-validation showed a mean absolute deviation of 3.45 years, with no significant sex difference	[25]
Body fluid/tissue source identification	*DACT1*, *USP49* (semen); *cg26107890*, *cg20691722* (differentiation between saliva and vaginal secretions)	Requires only trace amounts of DNA and does not cause excessive sample consumption	[26]
Inference of individual health-related phenotypes	*SDC2*, *TFPI2* (colorectal cancer); *NEUROG1* (feces); oral cancer methylation markers	Draws on disease-specific marker screening and validation frameworks to aid inference of health characteristics in individual cases	[27,28,29,30]

Note: ddPCR is the abbreviation for droplet digital PCR.

**Table 4:** Evaluation of the Maturity of Forensic Applications of Nanopore Sequencing Combined with Methylation Markers.

Application Type	Verification Stage	Key Considerations and Limitations
Age and Fluid Origin Inference	Analysis and Verification Phase	Attention should be paid to the group effect and the setting of evidence thresholds; inter-laboratory validation is still insufficient
Inference of individual health characteristics	Research Phase	The error rate is relatively high and the repeatability is insufficient; most of them are transferable methods, lacking forensic validation
Intricate mixed-sample deconvolution	Research Phase	The hybrid sample deconvolution algorithm is not yet mature, and the risk control of contamination needs to be optimized
Portable on-site rapid testing	Analysis and Validation Phase	The error rate and reproducibility are greatly influenced by the environment, and there is a lack of rules for determining the validity of evidence

## Data Availability

No new data were created or analyzed in this study. Data sharing is not applicable to this article.
